# Sexually transmitted infections and bacterial vaginosis and preterm birth in pregnant people living with HIV: A population-based cohort study

**DOI:** 10.1177/09564624251347458

**Published:** 2025-05-30

**Authors:** Jeffrey Man Hay Wong, Gal Av-Gay, Terry Lee, Arezou Azampanah, Chelsea Elwood, Julie van Schalkwyk, Laura Sauvé, Deborah Money

**Affiliations:** 1 Department of Obstetrics and Gynecology, 8166 University of British Columbia, Vancouver, BC, Canada; 2 Women’s Health Research Institute, 574117 BC Women’s Hospital and Health Centre, Vancouver, BC, Canada; 3 Centre for Advancing Health Outcomes, Vancouver, BC, Canada; 4 Department of Pediatrics, 8166 University of British Columbia, Vancouver, BC, Canada

**Keywords:** Human immunodeficiency virus, preterm births, sexually transmitted infections, bacterial vaginosis, pregnancy

## Abstract

**Background:**

While individual sexually transmitted infections are linked with preterm births, their synergistic impact among pregnant people living with HIV (PLWH) remain unclear. We aimed to identify the impact of antenatal sexually transmitted infections and bacterial vaginosis on preterm birth in PLWH.

**Methods:**

We completed a population-based cohort study using the British Columbia Perinatal HIV Surveillance Database, capturing all births in PLWH from January 1997 to December 2022. Univariate risk factors for preterm birth were identified using chi-squared tests, Fisher’s exact tests and t-tests, followed by a multivariate logistic regression analysis.

**Results:**

Of 578 singleton pregnancies, 111 (19.2%) had preterm births, of which 34 (31%) delivered before 34 weeks gestational age. In our population, 11% were identified with a sexually transmitted infection or bacterial vaginosis (STIBV) in pregnancy. The preterm birth rate in PLWH with antenatal STIBV was 37% compared to 17% in PLWH without STIBV (OR: 2.18; 95% CI (1.50 – 3.16); *p* = .0003). Preterm deliveries were more common in individuals with concurrent Hepatitis C (OR: 2.42; *p* < .0001), antenatal diagnosis of *Chlamydia* *trachomatis* (OR 2.17; *p* = .036), *Trichomonas* *vaginalis* (OR: 2.78; *p* < .001) and bacterial vaginosis (OR: 2.15; *p* = .003). After adjusting for ethnicity, history of preterm birth, substance use, concurrent Hepatitis C, CD4 count and viral suppression at delivery, STIBV remains an independent risk factor (OR: 2.09; 95% CI: 1.04 – 4.19; *p* = .039).

**Conclusion:**

Among PLWH, antenatal screening for sexually transmitted infections and bacterial vaginosis can identify individuals at the highest risk of preterm birth.

## Introduction

While many sexually transmitted and bloodborne infections are each correlated with preterm births,^1–3^ the synergetic impacts of co-infections in pregnant people living with human immunodeficiency virus (HIV) remain unclear. Over 20% of people living with HIV (PLWH) are found to have a co-infection with a genital tract infection in pregnancy.^4,5^ In the general population, sexually transmitted infections such as *Chlamydia trachomatis* (CT) and *Neisseria gonorrhoeae* (NG) are associated with preterm birth, premature rupture of membranes, postpartum infections, and neonatal infections such as pneumonia and ophthalmitis.^6–8^ In pregnant PLWH, co-infections with CT and NG have been associated with an increased risk of perinatal HIV transmission.^9,10^

In a recent meta-analysis, PLWH are at an increased risk of preterm birth compared to the general population.^11^ When examining only PLWH on combination antiretroviral therapy (cART), the elevated risk of preterm birth remains persistent.^12^ Factors that place PLWH at risk of preterm birth include co-infection with Hepatitis C, concurrent substance use, poor HIV control (lower CD4 count and unsuppressed viral load), and anemia.^13,14^ In the non-HIV population, meta-analyses have also shown a clear association between preterm birth and antenatal diagnosis of *Chlamydia trachomatis, Neisseria gonorrhoea*, *Trichomonas* *vaginalis* (TV), and bacterial vaginosis.^2,15,16^ However, in PLWH, only two studies examined CT and NG infections’ impact on preterm birth with conflicting results^4,5^ – while only one study studied the perinatal implications of TV.^5^

We conducted this study to understand our rates of sexually transmitted infections and bacterial vaginosis (STIBV) in pregnant PLWH and examine their impact on preterm birth rates in British Columbia, Canada.

## Methods

### Study population

We completed a prospective population-based cohort study on all pregnancies in PLWH in British Columbia, Canada. Our analysis was completed using the British Columbia HIV Perinatal Database, established in 1994. Singleton pregnancies from January 1, 1997 to December 31, 2022 were included in the analysis. Pregnancies beyond 1997 reflect a cohort where the use of antiretroviral therapy was standard in pregnancy. Exclusion criteria included multiple gestation pregnancies, spontaneous abortions before 20 weeks gestational age, therapeutic abortions, maternal deaths, and unknown gestational age at delivery. The conception and use of the database were prospectively approved by the University of British Columbia Children’s and Women’s Research Ethics Board (approval number H10-01186) and was supported through infrastructure from the Women’s Health Research Institute and BC Women’s Hospital and Health Centre. The British Columbia HIV Perinatal Database was established as part of the provincial surveillance program, where our research ethics board deemed that individual consent was not required.

### Data collection

All individuals living with HIV who become pregnant are followed directly or indirectly through the provincial perinatal HIV service and entered as part of provincial surveillance. Data, including early infant outcome data, is inputted routinely into a Research Electronic Data Capture (REDCap) database at the end of the pregnancy. Data sources include the British Columbia Antenatal Record, the provincial laboratory results repository, hospital records, and clinicians’ notes. Lack of antiretroviral therapy at delivery is defined as no antiretroviral therapy prescribed during the entire pregnancy. Hepatitis C diagnosis was determined by a positive Hepatitis C virus RNA (if available). If Hepatitis C IgG antibody is positive but RNA testing is unavailable, this would be also be determined as a Hepatitis C diagnosis. Diagnoses of bacterial vaginosis was determined by Nugent score > 6. For sexually transmitted and bloodborne infections, *Chlamydia trachomatis, Neisseria gonorrhoaea* and *Trichomonas* *vaginalis* infections were determined by positive polymerase chain reaction (PCR). Hepatitis B infections were deemed positive with a detectable Hepatitis B viral DNA (if available). If Hepatitis B DNA results were not available, a Hepatitis B diagnosis only based on HBsAg testing. Preterm births were defined as deliveries between 20+0 and 36 + 6 weeks gestational age. Information on the specific laboratory kits for each sexually transmitted and bloodborne infection is not available.

### Statistical analysis

All statistical analyses were conducted in R version 4.2.0 (Auckland, New Zealand). From a coding perspective, the combined STIBV variable represents at least one perinatal diagnosis of CT, NG, TV, and bacterial vaginosis, all established as risk factors for preterm deliveries in the general obstetrical population. The chi-squared and Fisher’s exact tests were used to complete univariate analyses on categorical variables. T-tests were conducted for continuous variables to determine statistical significance differences between preterm and term births. A multivariate logistic regression model was built to examine the association between STIBV and preterm deliveries while adjusting for risk modifiers with *p*-value < .1 in the univariate analysis, as well as clinically significant risk factors that have a *p*-value > .1. Due to significant number of missing data for body mass index, this was not included in the regression model. A *p*-value of less than 0.05 was used as the cut‐off for two‐sided significance.

## Results

Our provincial surveillance program included 889 pregnancies since its inception. After excluding pregnancies before 1997 (*n* = 127), therapeutic or spontaneous abortions (*n* = 127), multifetal pregnancies (*n* = 28), maternal deaths (*n* = 7), and unknown gestational age (*n* = 22), we analyzed 578 singleton pregnancies. Our preterm delivery rate in the cohort was 19.2% (*n* = 111), where 34 (31% of preterm births) were before 34 weeks gestational age.

Demographics and HIV-specific characteristics in our study population are described in Table 1. In our cohort, risk modifiers associated with preterm birth include ethnicity (*p* = .0002), substance use in pregnancy (*p* = .0004), failure to achieve virologic suppression (*p* < .0001), lack of antiretroviral therapy at delivery (*p* < .0001), and a delivery CD4 count < 350 cells/mm^3^ (*p* = .0003). The specific antiretroviral regimen taken in pregnancy was not correlated with preterm birth.

**Table 1.** Demographics of included cohort of people living with HIV in British Columbia.

		Cohort total (*n* = 578)	Term birth (*n* = 467)	Preterm birth (*n* = 111)	Odds ratio (95% CI)	*p*-value
		n (% of total)	n (rate of term birth)	n (rate of preterm birth)		
Age			31 ± 6	31 ± 5	0.99 (0.96, 1.03)	.68
BMI	<18.5	4 (2%)	4 (100%)	0 (0%)	N/A	.033
	18.5–24.9	87 (44%)	65 (75%)	22 (25%)	1	
	25–30	61 (30%)	51 (84%)	10 (16%)	0.65 (0.33–1.27)	
	>30	48 (24%)	45 (94%)	3 (6%)	0.25 (0.08–0.78)	
Ethnicity	Black	125 (22%)	115 (92%)	10 (8%)	1	.0002
	Indigenous	170 (29%)	122 (72%)	48 (28%)	3.53 (1.86–6.7)	
	White	193 (33%)	157 (81%)	36 (19%)	2.33 (1.2–4.53)	
	Other	90 (16%)	73 (81%)	17 (19%)	2.36 (1.13–4.91)	
Gravidity	1	89 (16%)	71 (80%)	18 (20%)	1	.65
	2	123 (22%)	97 (79%)	26 (21%)	1.05 (0.61–1.79)	
	3+	356 (63%)	293 (82%)	63 (18%)	0.88 (0.55–1.4)	
Parity	0	172 (31%)	133 (83%)	29 (17%)	1	.55
	1	152 (27%)	118 (78%)	34 (22%)	1.33 (0.85–2.07)	
	2	111 (20%)	93 (84%)	18 (16%)	0.96 (0.56–1.65)	
	3+	127 (23%)	103 (81%)	24 (19%)	1.12 (0.69–1.83)	
Previous preterm birth	Yes	83 (15%)	61 (73%)	22 (27%)	1.53 (1.02–2.3)	.066
	No	479 (85%)	396 (83%)	83 (17%)	1	
Gestational diabetes	Yes	54 (10%)	44 (81%)	10 (19%)	1.05 (0.58–1.89)	.85
	No	498 (90%)	410 (82%)	88 (18%)	1	
Hypertensive disorder of pregnancy	Yes	31 (6%)	25 (81%)	6 (19%)	1.06 (0.5–2.22)	.81
	No	524 (94%)	428 (82%)	96 (18%)	1	
Substance use in pregnancy	Yes	118 (20%)	81 (69%)	37 (31%)	1.95 (1.39–2.74)	.0004
	No	460 (80%)	386 (84%)	74 (16%)	1	
Combination ART regimen nearest delivery	Mono/Dual/ Triple NRTI	25 (5%)	22 (81%)	3 (12%)	0.68 (0.19–2.46)	.95
	NNRTI	90 (18%)	74 (82%)	16 (18%)	1.01 (0.43–2.36)	
	Protease inhibitors (PI)	121 (24%)	103 (85%)	18 (15%)	0.84 (0.36–1.96)	
	Boosted PI	241 (47%)	198 (82%)	43 (18%)	1.01 (0.47–2.19)	
	Integrase inhibitors	34 (6%)	28 (82%)	6 (18%)	1	
	Other	2 (0.3%)	2 (100%)	0 (0%)	0 (0 - NaN )	
On antiretroviral therapy at delivery?	Yes	535 (93%)	442 (83%)	93 (17%)	0.41 (0.27–0.60)	.0003
	No	42 (7%)	24 (57%)	18 (43%)	1	
Time on ART prior to delivery	<12 weeks	169 (29%)	114 (67%)	55 (33%)	1	<.0001
	>12 weeks	409 (71%)	353 (86%)	56 (14%)	0.42 (0.30–0.58)	
VL suppressed at delivery	Yes	425 (80%)	365 (86%)	60 (14%)	0.42 (0.29–0.59)	<.0001
	No	109 (20%)	72 (66%)	37 (34%)	1	
CD4 count at delivery	<350	123 (24%)	86 (70%)	37 (30%)	1	.0003
	>350	382 (76%)	326 (85%)	56 (15%)	0.49 (0.34–0.7)	

### Bacterial vaginosis, sexually transmitted and bloodborne infections

In our population, baseline incidence of co-infections and vaginal dysbiosis is as follows: Hepatitis B (*n* = 14; 2.5%), Hepatitis C (*n* = 83; 14.4%), *Treponema pallidum* (*n* = 5; 0.9%), CT (*n* = 20; 3.4%), NG (*n* = 4; 0.7%), TV (*n* = 22; 3.8%), and BV (*n* = 42; 7.3%). Table 2 compares the preterm birth rate between PLWH who had a co-infection or concurrent bacterial vaginosis in pregnancy compared to those who did not. A statistically significant difference in preterm birth was found in those who have concurrent Hepatitis C (OR: 2.42; 95% CI (1.72–3.39); *p* < .0001), an antenatal diagnosis of *Chlamydia trachomatis* (OR 2.17; 95% CI (1.23–3.81); *p* = .036), *Trichomonas vaginalis* (OR: 2.78; 95% CI (1.77–4.38); *p* = .0009) and bacterial vaginosis (OR: 2.15; 95% CI (1.40–3.29); *p* = .003). In amalgamating genital tract infections and bacterial vaginosis (i.e. STIBV), the preterm birth rate in PLWH with STIBV was 37% compared to 17% in those without STIBV (OR: 2.18; 95% CI (1.50–3.16); *p* = .0003). Risk factors for STIBV diagnosis in pregnancy include ethnicity (*p* < .001), unsuppressed viral load (*p* = .047), substance use in pregnancy (*p* < .001) and hepatitis C infection in pregnancy (*p* = .022) (Table 3). Treatment of the sexually transmitted infection before delivery occurred in 65% (13/20) of CT cases, 75% (3/4) of NG cases, 73% (16/22) of TV cases, and 55% (23/42) of BV cases. Untreated patients represented peripartum diagnoses, which did not have an opportunity for treatment. When comparing treated with untreated cases of STIBV, there are no differences in preterm births (40% vs 33%; *p* = .579).

**Table 2.** Sexually transmitted, bloodborne infections and bacterial vaginosis in pregnant people living with HIV.

		Cohort total (*n* = 578)	Term birth (*n* = 467)	Preterm birth (*n* = 111)	Odds ratio (95% CI)	*p*-value
		n (% of total)	n (rate of term birth)	n (rate of preterm birth)		
Syphilis	Yes	5 (1%)	3 (60%)	2 (40%)	2.1 (0.71–6.23)	.25
	No	573 (99%)	464 (81%)	109 (19%)	1	
Hepatitis B	Yes	14 (2%)	10 (71%)	4 (29%)	1.51 (0.65–3.51)	.32
	No	564 (98%)	457 (81%)	107 (19%)	1	
Hepatitis C	Yes	83 (14%)	51 (61%)	32 (39%)	2.42 (1.72–3.39)	<.0001
	No	495 (86%)	416 (84%)	79 (16%)	1	
Gonorrhoea	Yes	4 (1%)	2 (50%)	2 (50%)	2.63 (0.97–7.12)	.17
	No	574 (99%)	465 (81%)	109 (19%)	1	
Chlamydia	Yes	20 (3%)	12 (60%)	8 (40%)	2.17 (1.23–3.81)	.036
	No	558 (97%)	455 (81%)	103 (18%)	1	
Trichomonas	Yes	22 (4%)	11 (50%)	11 (50%)	2.78 (1.77–4.38)	.0009
	No	556 (96%)	456 (80%)	100 (18%)	1	
Bacterial vaginosis	Yes	42 (7%)	26 (62%)	16 (38%)	2.15 (1.40–3.29)	.003
	No	536 (93%)	441 (82%)	95 (18%)	1	
STIBV	Yes	65 (11%)	41 (64%)	24 (37%)	2.18 (1.5–3.16)	.0003
	No	513 (89%)	426 (83%)	87 (17%)	1

**Table 3.** Risk modifiers associated with antenatal diagnosis of STIBV in pregnant people living with HIV.

		Concurrent STIBV in pregnancy *n* = 65	No STIBV in pregnancy *n* = 513	Odds ratio (95% CI)	*p*-value
Age		30.5 ± 5.4	31.5 ± 5.6	0.97 (0.92, 1.01)	.16
Ethnicity	Black	9 (14%)	116 (23%)	1	<.001
	Indigenous	33 (51%)	137 (26%)	3.10 (1.43–6.75)	
	White	18 (28%)	175 (34%)	1.32 (0.58–3.05)	
	Other	5 (8%)	85 (17%)	0.76 (0.25–2.34)	
Viral load suppressed at delivery	Yes	44 (70%)	381 (81%)	0.55 (0.31–0.98)	.047
	No	19 (30%)	90 (19%)	1	
CD4 count at delivery	<350	20 (32%)	103 (23%)	1	.158
	>350	43 (68%)	339 (77%)	0.65 (0.37–1.16)	
Substance use in pregnancy	Yes	27 (41%)	80 (16%)	7.62 (4.40–13.18)	<.001
	No	38 (58%)	433 (84%)	1	
Hepatitis C infection	Yes	16 (25%)	67 (13%)	2.17 (1.17–4.04)	.022
	No	49 (75%)	446 (87%)	1	

### Multivariate analyses of PLWH and risk of preterm birth

After adjusting for ethnicity, history of preterm birth, perinatal substance use, concurrent Hepatitis C, protease inhibitor-based antiretroviral therapies, CD4 count and viral suppression at delivery (Table 4), STIBV in pregnancy remains an independent risk factor (OR: 2.09; 95% CI: 1.04–4.19; *p* = .039).

**Table 4.** Multivariate analysis for preterm birth in people living with HIV.

Risk modifiers	Odds ratio (95% CI)	*p*-value
Sexually transmitted infections or bacterial vaginosis	2.09 (1.04, 4.19)	.039
Unsuppressed viral load at delivery	2.04 (1.08, 3.84)	.028
Ethnicity		
Black	1	
Indigenous	2.56 (0.99, 6.65)	.054
White	2.90 (1.16, 7.26)	.023
Other ethnicities	2.33 (0.81, 6.73)	.118
History of preterm deliveries	1.51 (0.73, 3.09)	.263
Substance use in pregnancy	1.64 (0.86, 3.14)	.135
Hepatitis C co-infection	1.64 (0.83, 3.22)	.151
CD4 <350 cells/mm^3^ at delivery	1.68 (0.92, 3.09)	.093
Protease inhibitors based antiretroviral therapy	1.03 (0.57, 1.87)	.915

Number included in analysis: 456 patients.

## Discussion

Our 25-year cohort of PLWH in British Columbia has a preterm birth rate of 19.2% compared to the baseline preterm birth rate in the province of 7.4%.^17^ Eleven percent of pregnant PLWH were diagnosed with STIBV antenatally, and those with an antenatal diagnosis of STIBV were at higher risk of preterm births. It is particularly striking that although the rates are low in our population, individuals with trichomoniasis in pregnancy had a preterm birth rate of 50%. After adjusting for ethnicity, history of preterm birth, substance use, and HIV suppression, STIBV remains an independent risk factor for preterm birth.

The prevalence of antenatal sexually transmitted infections in our British Columbia cohort is lower compared to other populations living with HIV. In contrast, the US-based Pediatric HIV/AIDS Cohort Study established higher rates of sexually transmitted infections in pregnancy: CT (7.7%), NG (2.3%), TV (14.5%) and syphilis (2.4%).^5^ Despite our consistently lower rates compared to the United States, our cohort’s prevalence remains higher than that of the general Canadian pregnant population. The only published Canadian study estimated CT and NG prevalence in the general pregnant population at 2.0% and 0.2%, respectively.^18^ The incidence of positive prenatal syphilis testing is 0.03% in British Columbia.^19^ No Canadian studies have evaluated TV prevalence or incidence in pregnancy. Given the higher incidence of infections in PLWH compared to the general population, our study reinforces the importance of comprehensive sexually transmitted and bloodborne infection screening in pregnancy in this population.

Global preterm birth rates for PLWH range between 5.2% and 73.0%,^11^ while the preterm birth rate in our study is 19.2%. More importantly, our cohort’s preterm birth rate is much higher than the provincial rate of 7.4%.^17^ Only two other studies of PLWH have examined the correlation between sexually transmitted infections and preterm labour with conflicting results.^4,5^ The US-based Pediatric HIV/AIDS Cohort Study did not find a difference between CT, NG, and TV diagnoses and preterm births.^5^ Conversely, the International Maternal Pediatric Adolescent AIDS Clinical Trials Network P1043 trial identified that PLWH with a diagnosis of CT and NG have a higher preterm birth rate of 29%, compared to their cohort baseline of 11%.^4^ However, this sub-study recruited PLWH at delivery, completed intrapartum testing for CT/NG and excluded PLWH < 32 weeks gestational age. Our higher rates of preterm birth likely reflect capturing preterm deliveries before 32 weeks gestational age and the impact of antenatal diagnosis. Given the significant overlap between STIBV and social determinants of health, our study uniquely demonstrates that STIBV remains an independent risk factor after adjusting for ethnicity, substance use, CD4 count, Hepatitis C co-infections, antiretroviral therapy regimens, viral suppression, and preterm birth history.

PLWH are at increased risk of bacterial vaginosis acquisition,^20^ and its prevalence reaches up to 47%, depending on the region and population.^21^ Only 7.3% of pregnant PLWH in our population were identified with bacterial vaginosis. Currently, the Society of Obstetricians and Gynaecologists of Canada does not recommend routine screening for BV in pregnancy for asymptomatic individuals without risk factors for preterm birth.^22^ Testing is only recommended for pregnant people with symptomatic bacterial vaginosis. Given this, our low rate reflects the incidence of symptomatic bacterial vaginosis for pregnant PLWH. However, our study highlights that when clinically indicated swabs are completed, people living with HIV with a positive finding of BV are at higher risk of preterm births.

The 25-year duration of our provincial surveillance program is both a strength and a limitation for our study. While our analysis provides a comprehensive perspective of our cohort of PLWH in British Columbia, clinical care has evolved significantly over the years. Diagnostic testing for sexually transmitted infections has improved over the years with increased implementation of nucleic acid amplification testing across our province. Additionally, our infection and bacterial vaginosis cases reflect individuals with positive test results but do not identify the proportion of individuals not screened during pregnancy. As such, we expect the actual rate of concurrent infections to be higher than our reported rate. This is particularly true for conditions not included in the routine prenatal screening, such as trichomoniasis and bacterial vaginosis. Our study is also underpowered to detect whether treatment of STIBV reduces preterm delivery. In a cohort study like ours, individuals who receive treatment during pregnancy may represent individuals with better access to healthcare, which can also impact preterm delivery rates. British Columbia is also currently struggling with a concurrent substance use epidemic, which is reflected in our cohort’s demographics. The syndemic of HIV and substance use impacts prenatal care and contributes to our high preterm delivery rate of 19.2%. Despite our attempts to adjust through the multivariate analysis, the prevalence of substance use and associated social determinants of health may not reflect the global population of pregnant PLWH, which may limit the external validity of our results.

In conclusion, our analysis demonstrates that pregnant PLWH have high rates of concurrent sexually transmitted and bloodborne infections, as well as bacterial vaginosis. While PLWH are already at increased risk of preterm births, those who have been identified with STIBV in pregnancy are at even greater risk. Due to high concurrent rates of infections and bacterial vaginosis, this study reinforces the need for comprehensive screening in pregnancy for people living with HIV.

## Data Availability

The datasets generated during and/or analyzed during the current study are available from the corresponding author on reasonable request.*
